# A Rare Variant and Unusual Presentation of Holt Oram Syndrome in a Child

**DOI:** 10.7759/cureus.31076

**Published:** 2022-11-04

**Authors:** Rasagnya M Reddy, Mahaveer S Lakra, Revat J Meshram, Amar Taksande, Mayur B Wanjari

**Affiliations:** 1 Department of Paediatrics, Jawaharlal Nehru Medical College, Datta Meghe Institute of Medical Sciences, Wardha, IND; 2 Department of Pediatrics, Jawaharlal Nehru Medical College, Datta Meghe Institute of Medical Sciences, Wardha, IND; 3 Department of Research, Jawaharlal Nehru Medical College, Datta Meghe Institute of Medical Sciences, Wardha, IND

**Keywords:** skeletal manifestations, supraventricular tachycardia, radial ray defect, atrial septal defect, holt oram syndrome

## Abstract

Holt Oram syndrome is a rare genetically inherited disorder characterized by various skeletal abnormalities of the upper limb with an underlying structural heart defect. Family history and conduction defects may or may not be there. The diagnosis is often clinical; if the criteria are not fulfilled, then genetic studies may be required. This syndrome can also occur with complex congenital heart defects, heart blocks, and arrhythmias. The correlation between the severity of cardiac and skeletal manifestations was not studied, and in severe conditions and acute presentation, the patient may have atrial, supraventricular, and ventricular fibrillation. The association of radius with hand deformity and the cardiac defect is well reported, but this disease's involvement of the humerus and Supraventricular tachycardia (SVT) is rare. Here, we are reporting a case of Holt Oram syndrome in a five-year male child with unusual skeletal hypoplastic humerus who presented with breathlessness, palpitations, and supraventricular tachycardia and suffered a total of three episodes which were managed with adenosine and cardioversion successfully. The involvement of the humerus, along with symmetrical bilateral radial defect with Atrial septal defect (ASD) and Supraventricular tachycardia (SVT), is a unique feature of Holt Oram syndrome seen in our case.

## Introduction

Holt Oram syndrome is rare and genetically inherited, having an autosomal dominant pattern with a prevalence rate of 0.95/100,000 births [1]. It is also called heart hand syndrome, and many phenotypes have been reported. The criteria for diagnosing this syndrome are abnormalities in the radius in at least one upper limb and congenital heart disease with or without conduction diseases in the heart [2]. The most common heart diseases associated with this syndrome are atrial septal defects (ASD), ventricular septal defects (VSD), and cardiac conduction defects [3]. Cases have also been reported with other congenital cyanotic heart diseases like tricuspid atresia and transposition of great arteries [4].

Sometimes patients may present with arrhythmia, heart failure, severe pulmonary hypertension, and severe heart block. Most of the skeletal abnormalities in holt Oram syndrome are seen in the form of radial ray abnormalities, hypoplastic radius/ulnar in either the upper limb or both the upper limbs, triphalangeal abnormalities, metacarpals abnormalities, and finger-like thumb. The distribution of anomalies might be bilateral or unilateral and asymmetrical or symmetrical [5].

Here, we report a different variant and unusual presentation of this disease in a child presenting with unusual distinctive skeletal malformations in the form of hypoplastic humerus along with the bilateral absence of radius in both upper limbs, hand anomalies, underlying congenital heart disease, who presented in emergency with supraventricular tachycardia, which is a rare association in Holt Oram syndrome. Though radius is often involved in Holt Oram syndrome, a hypoplastic humerus is a rare entity, and very few cases have been reported in the literature.

## Case presentation

A five-year-old male child, a product of non-consanguineous marriage, presented with complaints of increased work breathing and increased fatiguability for 15 days. History of a sudden increase in palpitations and difficulty in breathing for two days. The patient had a significant history of neonatal intensive care unit (NICU) stay for three days in the neonatal period, in view of having respiratory distress, and received treatment in the form of continuous positive airway pressure (CPAP) and oxygen by prongs. The patient was having significant skeletal abnormalities in the upper limb of both hands and some underlying cardiac anomalies also. The baby was managed accordingly and was advised to be in follow-up. The developmental history was normal, and intelligence and the scholastic performance were good. There was no significant family history of similar skeletal anomalies or heart disease in any of the family members.

**Figure 1:**
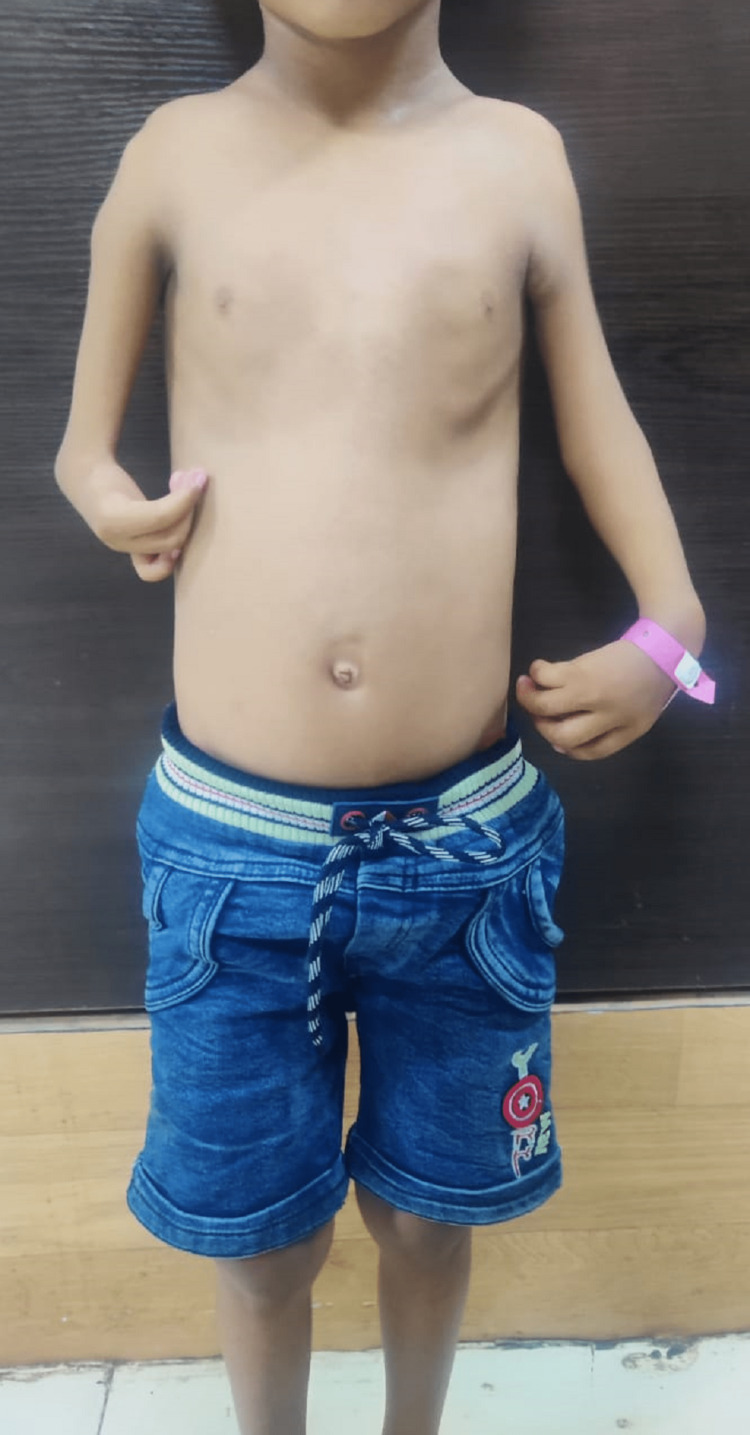
Shows Both the Hands Were Incurved and Pronated with the Discrepancy in Bulk, Posture and Digital Shortening with Radialisation of Thumb

On examination, the patient was conscious, anxious, breathless, afebrile, with Heart rate of 190 beats per minute, respiratory rate was 28 per minute with increased work of breathing, spO2 92 percent with oxygen, peripheral pulses well felt, and blood pressure 94/68 mm of Hg, with no cyanosis. On skeletal examination, there were abnormal shortening and deformity were present in the upper limb. The right arm was small, measuring 6 cm, and the forearm was longer as compared to the left arm. The arm of the left limb was 4.5 cm longer than the right limb and had flexion deformity and abnormalities of the thumb and fingers in both hands. Both the hands were incurved and pronated with the discrepancy in bulk, posture, and digital shortening with radialisation of thumb as shown in (Figure 1). On cardiovascular examination, the apex beat was in the fifth intercostal space; there was no thrill or diastolic shock. On auscultation, tachycardia was present, and a grade 3 ejection systolic murmur was noticed in the pulmonary area. Respiratory system examination showed bilateral equal air entry and a clear chest. On per abdomen examination, the abdomen was soft, and the liver and spleen were not palpable. Laboratory investigations were showing Hb 12.5 gm, total leucocyte count 12000/cumm, platelet 2.5 lakhs, SGOT 44 IU/L, SGPT 42 IU/L, ALP 112 IU/L, total bilirubin 0.8 mg/dl, serum creatinine 0.8 mg/dl, urea 22 mg/dl, serum sodium 138 meq/l, and potassium 4.1meq/l.

**Figure 2:**
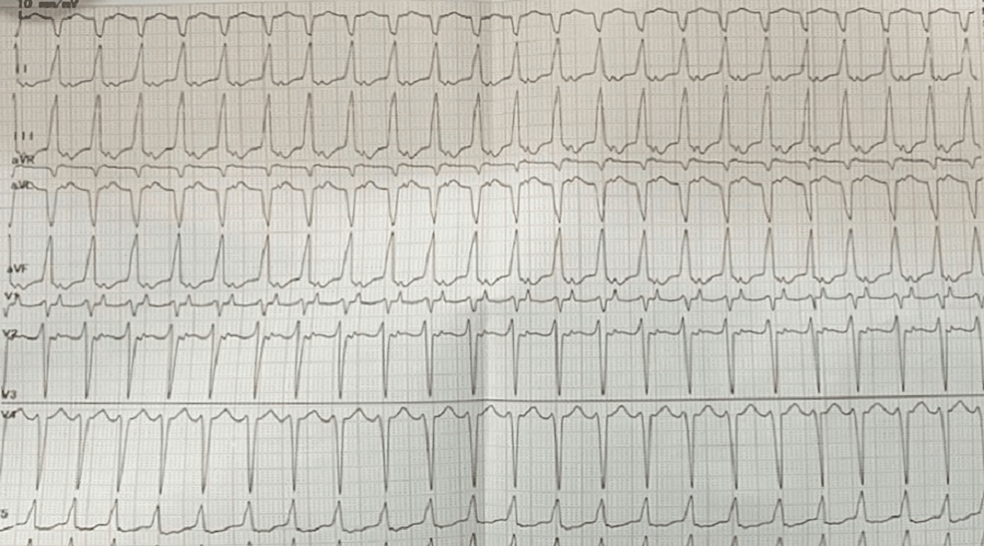
Showing HR of 194 with Absence of P-Wave, Narrow QRS Complex and Supraventricular Tachycardia.

**Figure 3:**
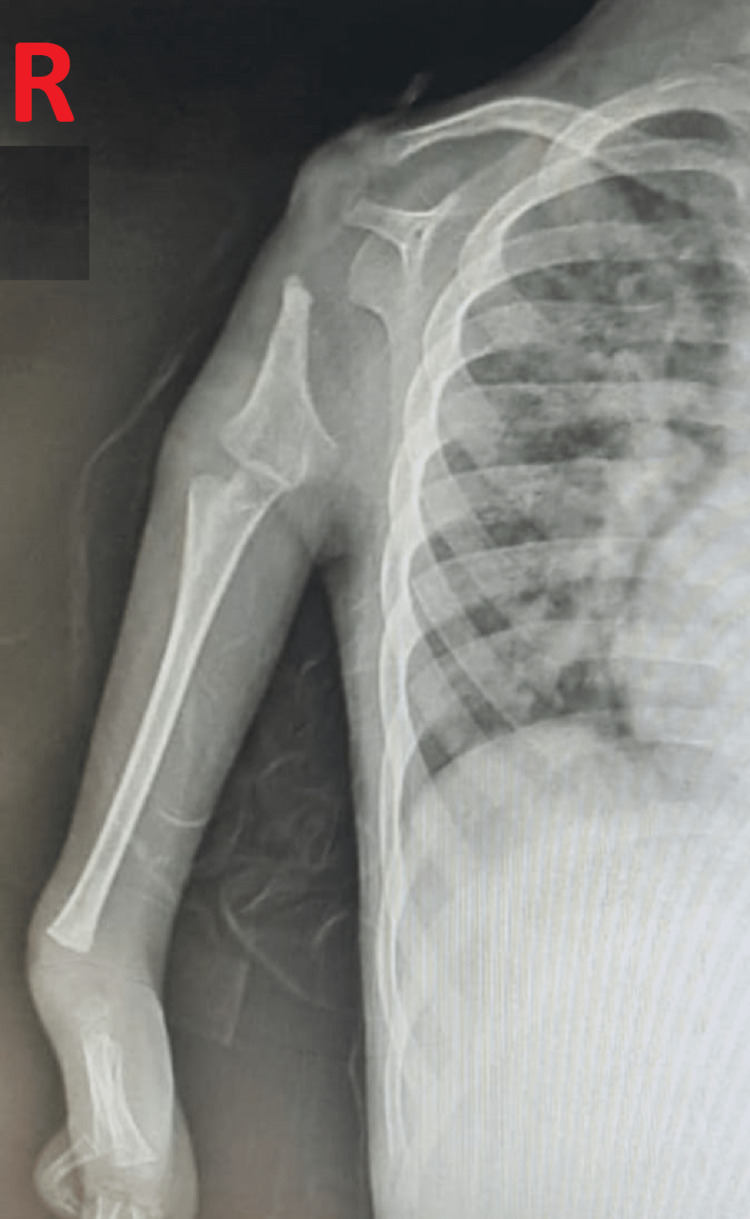
Skeletal Abnormalities in Form of Abnormal Incurving of Hand and Absence of Radius Bone.

**Figure 4:**
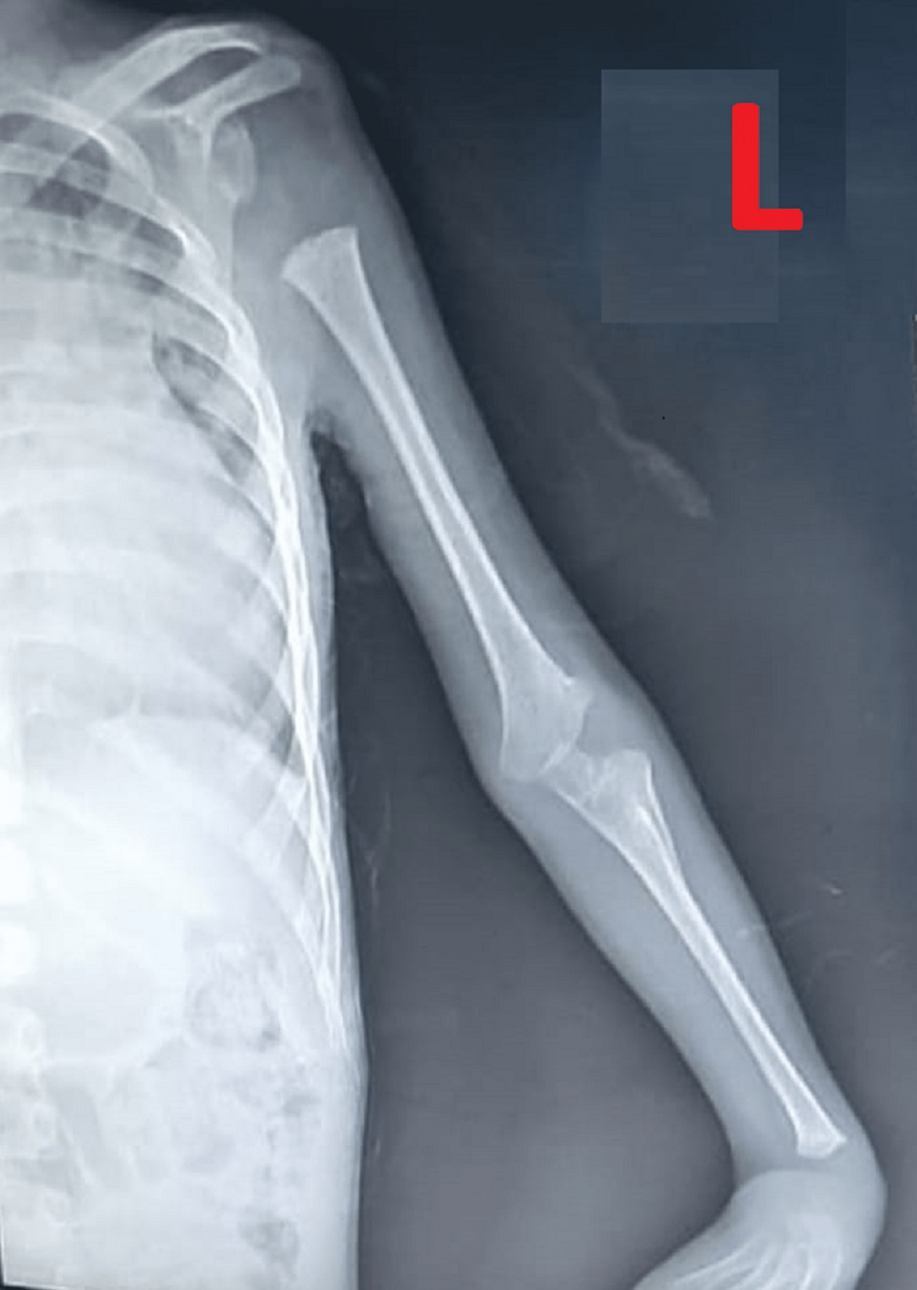
X-ray of Right Limb Showing Skeletal Abnormalities Inform of Hypoplastic Humerus and Absence of Radius With Metacarpal Anomalies

The patient was stabilized with IVF, oxygenation, and respiratory support, and frusemide and injection dobutamine were started. ECG was done showing HR of 190 beats per minute with the absence of p-wave suggestive of supraventricular tachycardia, as shown in Figure 2. The patient was given an injection of adenosine 0.1 mg by IV push stat after trying other maneuvers but required a repeat dose also. The patient had a total of three episodes of SVT during a hospital stay, which was managed successfully with an injection of adenosine, followed by cardioversion. X-ray of the upper limb was done, which was showing a bilateral absence of radius, and hypoplastic humerus on the right side along with metacarpal abnormalities of the hands, as shown in (Figures 3, 4). Chest X-ray was done s/o multiple small opacities present on right perihilar with mild pneumonitis. 2D echocardiography was showing ASD 7 mm with bilateral flow through the defect with dilatation of the right atrium without pulmonary hypertension, as shown in Figure 5. No other structural cardiac anomalies were found on the echo. Ultrasonography of the abdomen was done, which was normal. The diagnosis of Holt Oram syndrome with supraventricular tachycardia was made based on the clinical criteria, as laid down in the literature. The genetic testing could not be done because of the financial constraints and the non-availability of tests in our Institute. The child was managed successfully and discharged from the hospital on antiarrhythmic medication. An orthopedic opinion was sought, and conservative management was advised. The patient was reviewed after 15 and 30 days, respectively, and was found to be doing well. For ASD cardiac surgery was planned after three months, and the patient was put on infective endocarditis prophylaxis.

## Discussion

Holt Oram syndrome is also called atrio-digital dysplasia and is an autosomal dominant condition with rare occurrence. It is characterized by the expression of various skeletal abnormalities of the upper limb and congenital heart diseases with or without conduction abnormalities. Holt-Oram syndrome was first defined by Mary Holt and Samuel Oram in a family where four generations were suffering from heart defects with abnormal conduction defects and skeletal deformity of the hand in 1960 [6]. The Holt Oram syndrome should be suspected if it fulfills the following clinical criteria (Table 1).

**Table 1:** Clinical Criteria of Holt Oram syndrome ASD: Atrial septal defect, VSD: Ventricular septal defect

Sr. No.	Clinical Criteria
1	If any individual is presented with upper limb anomalies involving carpal, thenar or radial bones, the anomalies may be unilateral or bilateral symmetrical or asymmetrical, which is confirmed by the X-ray, as defined by Poznaski et al. [7] and Basson et al. [8] in their criteria.
2	Presence of underlying congenital heart diseases like ASD or VSD.
3	Presence of any type of conduction defect mostly A-V blocks, if no structural heart defect.
4	The significant family history of congenital heart disease and conduction defect in a first-degree family relative may or may not be there
5	Confirmed by genetic testing [9]

Gholarmerza Nourdzad et al. [10] reported a similar case of Holt Oram syndrome with atrial and ventricular septal defects with incomplete growth of clavicle. The patient had an absence of radius in both the upper limbs, like our index case. In our case, the patient had significant limb length disproportion along with hypoplastic humerus, bilateral symmetrical absence of radius, a structural heart defect, and supraventricular tachycardia, which is an abnormal presentation in Holt Oram syndrome. The involvement of the humerus and SVT is a rare clinical presentation of Holt Oram syndrome, which is a unique feature seen in our index cases [7,8,11].

The possible mechanism has been explained by the involvement of the limb with the heart during heart development because of some mutation of genes. This syndrome does not involve the lower limb because the embryogenesis occurs in the fourth to fifth week when the lower limbs are not formed, so the involvement of the lower limb rules out Holt Oram syndrome [5,8]. Diagnosis of Holt Oram syndrome is generally made by the clinical presentation of limb anomalies, preaxial radial X-ray, and significant personal and family history of cardiac and conduction defects. If the clinical features are clearly evident, then the genetic molecular study is diagnostic. It may be a single gene testing of TBX5 or multiple genetic panel testing. In this syndrome, TBX5 gene function is affected, and it is involved in about 75% of the cases. Most of the cases run in families; however, 40%-65% of cases may have spontaneous mutation and sporadic [8,9].

Mohmmad Ilyas et al. [12] reported a case of Holt Oram syndrome with metacarpal bone abnormalities and other associated cardiac defects. A similar case was also reported by Vipan Garg et al. [13] in a neonate who presented with Holt Oram with congestive failure, and Gupta M et al. [14], who incidentally diagnosed a 40 years old patient with varied upper limb anomalies. In our case, humerus, radius, and carpal bones were involved.

The management includes a multidisciplinary team approach involving the pediatrician, orthopedics, physiotherapist, hand surgeon for reconstructive surgery, and a genetic specialist. The main aim is rehabilitation, and to restore function, and, if possible, to do reconstructive surgery [2]. Pharmacological management for pulmonary hypertension and heart block may be needed, and heart surgery can be offered for significant lesions. An opinion from a cardiologist may be taken for the need of starting anticoagulant, and antibiotic prophylaxis and for the prevention of complications and prophylaxis of infective endocarditis. The patient’s condition should be followed up to assess the risk, and continuous monitoring at follow-up is required. The genetic testing of families should also be offered and must be screened for structural and conduction anomalies [8,9].

## Conclusions

Holt Oram syndrome is a rare disease multisystem disease involving the upper limb and heart and having a conduction defect. The presentation may vary and can have varied bony involvement. Any child presenting with skeletal anomalies must be evaluated for underlying heart defects and conduction anomalies. The patient may have an acute presentation in the form of arrhythmia requiring immediate intensive care. A multidisciplinary team is a strategy for further management.
